# The Parallel Consultation: A Literature Review of Patient Use of Large Language Models and Its Implications for Psychiatric Clinical Decision-Making

**DOI:** 10.7759/cureus.115382

**Published:** 2026-08-28

**Authors:** Aryan Kahlon, Lauren L Wallace, Tazmihl Walker, Shanu Sivakumar, Bani Brara, David G Bawden

**Affiliations:** 1 Medicine, St. George's University School of Medicine, True Blue, GRD; 2 Medicine, Trinity School of Medicine, Ribishi, Saint Vincent and the Grenadines, USA; 3 Psychiatry, AdventHealth Glen Oaks, Glendale Heights, USA

**Keywords:** ai chatbot, artificial intelligence (ai), clinical decision-making, generative artificial intelligence, large language model (llm), mental health assessment, mental health chatbot, mental health provider, psychiatry and mental health, self-disclosure

## Abstract

Large language models (LLMs) are immensely popular, being adopted by the general public for information and assistance in tasks, and increasingly in mental health support. This is especially true at a time when psychiatric care is limited by cost, availability, and stigma. More patients are using generative artificial intelligence (AI) for mental health guidance without professional medical oversight, and without disclosing usage to practitioners. This creates a clinical blind spot in which there lies a gap between what the practitioner knows and what has already altered the patient’s presentation. Undisclosed AI use can undermine the trust-building cycle in a clinical relationship, as patients may defer trust to a source the clinician is not aware exists, while arriving with AI-generated interpretations that might differ from clinical judgment. This narrative literature review synthesizes emerging evidence of how patient use of LLMs can complicate psychiatric clinical decision-making to raise awareness among mental health practitioners. Patients report utilizing AI to augment or substitute for therapy due to cost and accessibility, and finding the experience to be validating and non-judgmental. Having received potentially biased or inaccurate AI-generated information, patients may present with AI-generated self-diagnoses, altering symptom framing and affecting treatment adherence and the therapeutic relationship. Daily interactions with AI chatbots have been reported to coincide with delusional thinking, contributing to an emerging phenomenon of AI-associated psychosis, putting patients further at risk. The variation in the types of AI chatbots also raises safety concerns, including hallucination, sycophancy, and model bias. These risks dramatically change how a patient may present themselves in a clinical encounter. To screen for AI use at intake as well as practitioner education to recognize sycophancy and other AI-associated risks, frameworks developed by professional societies are all needed. Prospective research designs to study the tools patients use are lacking. The clinical reality is that patients and technology are both outpacing the clinical training and guidelines needed to address this growing challenge.

## Introduction and background

Artificial intelligence (AI), more specifically, large language models (LLMs), has rapidly become popular among the general public for searching for information, work, news, and physical exercise [1]. According to a 2026 Pew Research Center survey of the general public, from 33% in 2023, 49% of Americans now use AI chatbots [1]. Of these respondents, 24% consult chatbots daily [1]. In marketing, AI has been invited into homes through products such as smartwatches that track steps, sleep, and other health data, as well as smart doorbells that remain ever vigilant and safe [1]. LLMs could seem popular as a novelty during their introduction, as they can effectively understand and build language from publicly available datasets to convey ideas or meet the requests of the user [2]. LLMs present as increasingly sophisticated with each model release, reinforcing a public narrative of surpassing human performance in communication, learning, and reasoning [3]. These fundamentals have huge repercussions in the medical space, more specifically, if and when the user decides to trust their LLM as a confidant and/or mental health provider.

Multiple surveys have shown that there is an increase in the use of LLMs specifically for medical advice and emotional support reported in studies as early as 2021 [1-4]. Furthermore, as the user base of LLMs continues to increase, it is reasonable to infer that more people will turn to LLMs for mental health support rather than seeking out existing mental health resources [2-4]. From the perspective of an average LLM user, there are several advantages to using an AI chatbot to solicit advice and feedback. With current healthcare systems where psychiatric appointments can take several weeks to book, and where cost remains a barrier for consistent, quality mental healthcare, generative AI chatbots offer something the conventional system cannot: immediate, stigma-free access to a conversational partner at any hour, at little cost [3,4]. Due to their training over vast amounts of information on the internet, LLMs can continually offer new insights, advice, and feedback, all tailored to the user’s requests, delivered in a matter of seconds [4].

In the context of mental health, the importance of how the user may perceive their version of the LLM emotionally, or in other words, their compatibility, cannot be overstated [3,4]. In an interview conducted in 2024 with those who admitted to utilizing generative AI chatbots for mental health support, 19 participants (all over the age of 16, without geographic restrictions) reported their experiences as positive overall [3]. The same study reported that participants perceived chatbots as creating a non-judgmental atmosphere, validating their concerns and providing advice that at times can be considered “life-changing” [3]. In another study, an online survey conducted in 2026 among the general population of US adults, representing a mean (standard deviation) age of 47.3 (17.1) years old, with 20,847 participants, 10.3% reported daily use of AI. Another notable finding within this study with survey-weighted regression models was that more frequent use of AI was associated with a greater risk of moderate depression by a factor of 30% [5]. This study also highlighted that adverse mental health is more prominent among young adults compared to older cohorts [5]. These studies highlighted the growing number of users turning to LLMs for mental health support, which increases the need to study these relationships. These experiences of validation and emotional reliance among some individuals with mental health concerns using LLMs collectively shape a reality that the treating practitioner may be entirely unaware of.

For a medical practitioner, the implications of patients using AI for guidance and support in the context of any mental disorders extend directly into the clinical encounter. AI is actively influencing patient behavior and medical decision-making [4]. Companies whose products are LLMs explicitly state that guardrails are set up in the form of protocols if users demonstrate behavior deemed concerning, either flagging the user to staff or referring the user to seek professional help [3]. At the surface, this would appear to promote responsible use; however, interviewees reported these guardrails as disrupting their emotional sanctuary, even as perceiving said guardrails as a rejection at their lowest point [3]. From this same study, one interviewee reported that upon encountering guardrails with their LLM, they would begin to self-censor, something other studies seem to corroborate in terms of behavior illustrated by similar users [3,6]. Overall, these findings point toward a newly developing clinical picture in psychiatry: the question is no longer whether patients are using AI for mental health support, but rather how practitioners should respond when they do.

Regardless of a medical professional’s training, the psychiatric well-being of their patient is paramount, a standard that is increasingly becoming more complex due to the ever-increasing use of AI. The 2026 survey of the American Psychological Association reported that 77% of 1,200 psychologists have treated patients who have used AI for mental health support [7]. Of the same cohort, 30% of psychologists reported that their patients had used AI to self-diagnose before seeking professional help [7]. AI use is not yet a standard question upon intake, and studies confirm that patients rarely self-disclose their chatbot use to their provider [6]. This compounding silence is what worsens the clinical blind spot. While there is broad agreement on the urgency of updating clinical frameworks, this review synthesizes evidence from multiple sources to bring into focus a clinical reality that practitioners can no longer afford to overlook [2,4,6].

## Review

Literature search

This study is a narrative review of the available literature on patient perceptions, safety, disclosure, clinical approaches, and outcomes associated with artificial intelligence (AI) use in the context of mental health support. Given the rapid evolution of generative AI capabilities, the review’s synthesis approach remained narrative rather than systematic; however, search and eligibility criteria are reported in full below to support transparency and reproducibility.

Evidence was synthesized narratively rather than through a quantitative or algorithmic process. Because this is a narrative rather than a systematic review, studies were prioritized for inclusion based on direct relevance to the review’s clinical questions, recency, and methodological quality, rather than a formal scoring system. To limit selection bias, eligibility criteria were finalized before full-text review, and inclusion decisions were cross-checked among the screening authors.

A literature search was conducted across three databases (PubMed, PsycINFO, and Google Scholar), covering publications from January 2020 to July 2026. Each database was first accessed in late July 2026 and searched using the following terms: “use of AI in psychiatric patients,” “patient medical decision making by AI,” “AI chatbot for mental health,” “LLM disclosure to psychiatrist,” “LLM created psychosis,” “LLM complications and safety concerns,” and “LLM vs CBT.” Databases were last accessed in August 2026.

Studies were included if they were peer-reviewed, full-text publications, and published in English, and addressed patient perceptions, safety, disclosure, or clinical outcomes of AI use in mental health support within the specified date range. Preprints were included on an exceptional basis where no peer-reviewed equivalent was available, given the current pace of publication in this field at the time of the search. Studies were excluded if full text was not available, if they were not peer-reviewed (aside from the preprint exception above), if they were duplicate publications or letters to the editor, or if they were not written in English. Google Scholar does not offer a peer-reviewed filter; accordingly, results were screened at title and abstract stage, and only those published in identifiable peer-reviewed journals were taken forward. Full texts of Google Scholar results were retrieved directly from journal websites or PubMed Central rather than through Google Scholar’s native links, to ensure the published version of record was assessed.

As seen in Figure 1, 786 full-text articles were identified across three databases for eligibility. Following title and abstract screening, 63 publications were assessed in full text, of which 30 met eligibility criteria. Combined with two supplementary sources identified through institutional website searches, a total of 32 publications were included in the final synthesis. Study types included systematic reviews with and without meta-analyses, narrative reviews, qualitative studies, cross-sectional studies, case reports, commentaries, a retrospective cohort study, and a mixed-method survey. Additionally, an institutional report and a professional society report were considered to add further context. Greater weight was given to systematic reviews and meta-analyses for quantitative and generalizable claims. In comparison, case reports and commentaries served as expert opinion and emerging clinical examples rather than primary evidence. Six authors independently screened all articles and abstracts for relevance and eligibility; disagreements were resolved by group discussion and consensus in conjunction with a senior author.

**Figure 1:**
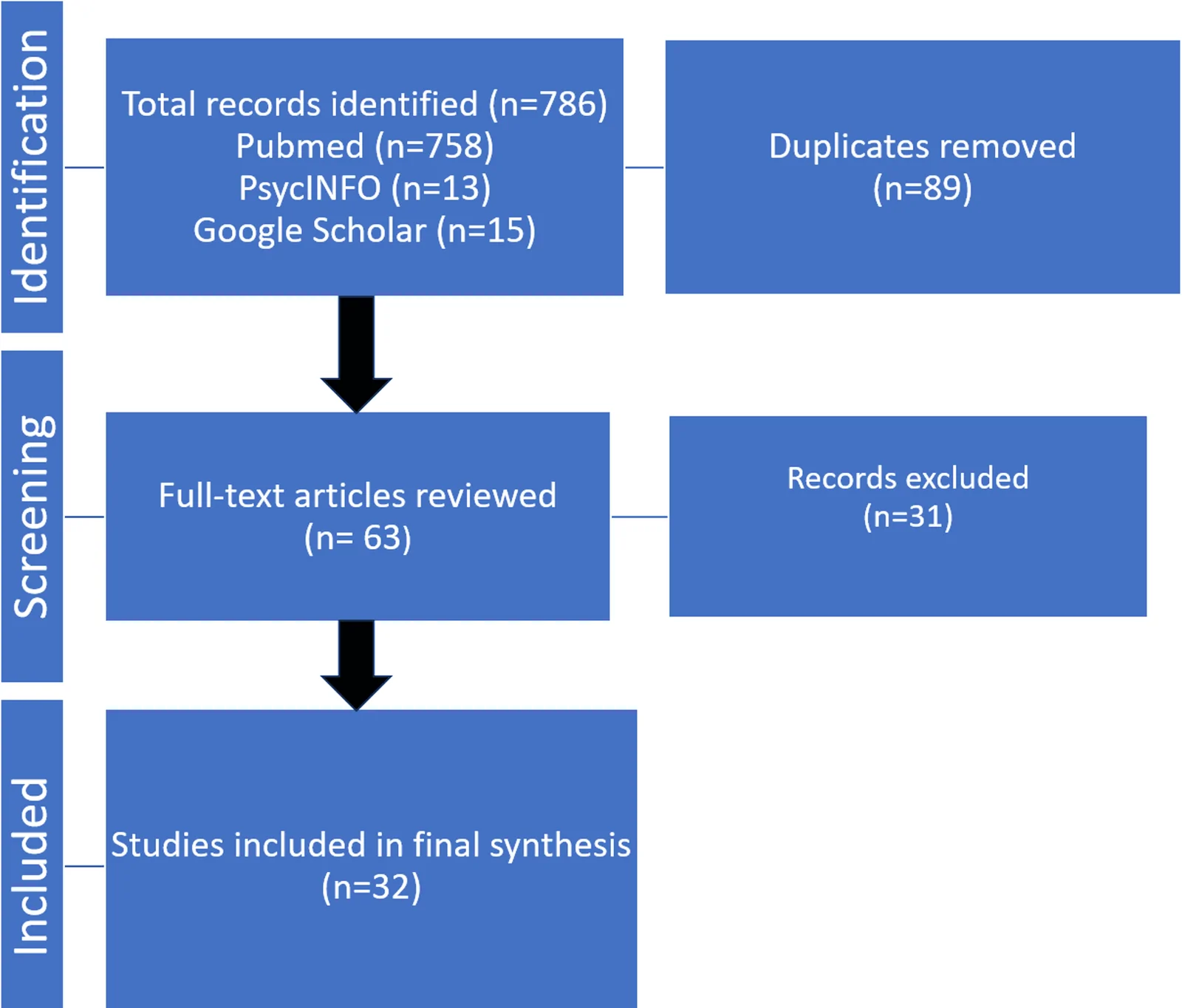
Flowchart illustrating the identification, screening, and inclusion process for studies evaluating relationships and the use of artificial intelligence chatbots in the context of mental health support Made using PowerPoint SmartArt graphic

Perception of AI treatment among its users

For any medical practitioner, encountering a patient whose medical decision-making is influenced by AI warrants an investigation into the extent of this influence. At this time, our understanding in this area is limited. With the few studies and interviews available currently, generative AI users admit to using their LLMs to either augment their therapy sessions or substitute for therapy entirely [3,8]. Furthermore, in a separate study, users have self-disclosed on a social media site (Reddit) managing mental health issues such as obsessive-compulsive disorder, post-traumatic stress disorder, anxiety, and eating disorders with generative AI [8]. Regarding substituting a human therapist with generative AI, it is worth noting that one subject of an interview explained that, due to cost and availability, seeking a human therapist was simply not feasible, whereas another subject explicitly stated that therapy with a psychologist was inadequate to the answers they were seeking [3]. A separate analysis of social media posts documented users who prompted their LLMs to employ strategies or simulate treatments, such as cognitive behavioral therapy (CBT) or dialectical behavior therapy [8]. Several participants in an interview have stated that generative AI chatbots have helped them heal from mental stress and trauma through the generation of imagery relevant to their situation or engaging in role play to offer new perspectives [3].

Users from multiple interviews have explicitly stated that they view their LLMs as a support to their mental well-being and, at times, as a trusted companion, one that validates their concerns and, most importantly, makes them feel seen or heard [3,8]. There are recurrent themes underlying these experiences as to why these treatments work with users. LLMs are easily accessible with regard to cost; they are always available and offer validating advice within seconds [4,8]. LLMs are perceived as free of judgment, creating an environment where users feel safe to share their most intimate thoughts and trauma [3,8]. LLMs are perceived to have exceedingly high intelligence thanks to quickly reading available information online and disseminating it for the user to understand more easily. This is paired with LLM marketing exhibiting passing multiple professional examinations or making accurate diagnoses and efficient problem-solving across multiple fields [3,4,8]. These themes can offer a glimpse of understanding why patients may turn to generative AI for help rather than pursue traditional mental health resources. Despite what can be interpreted at the surface as perceived efficacy of generative AI, multiple respondents have stated that generative AI still falls behind when it comes to taking initiative in discussions or employing real empathy [3,8]. The depth of engagement described across these accounts of emotional reliance, therapeutic simulation, and a sense of being heard represents a clinical blind spot, as seen in Figure 2, that rarely surfaces in the consultation room unless practitioners actively create the conditions for it to do so.

**Figure 2:**
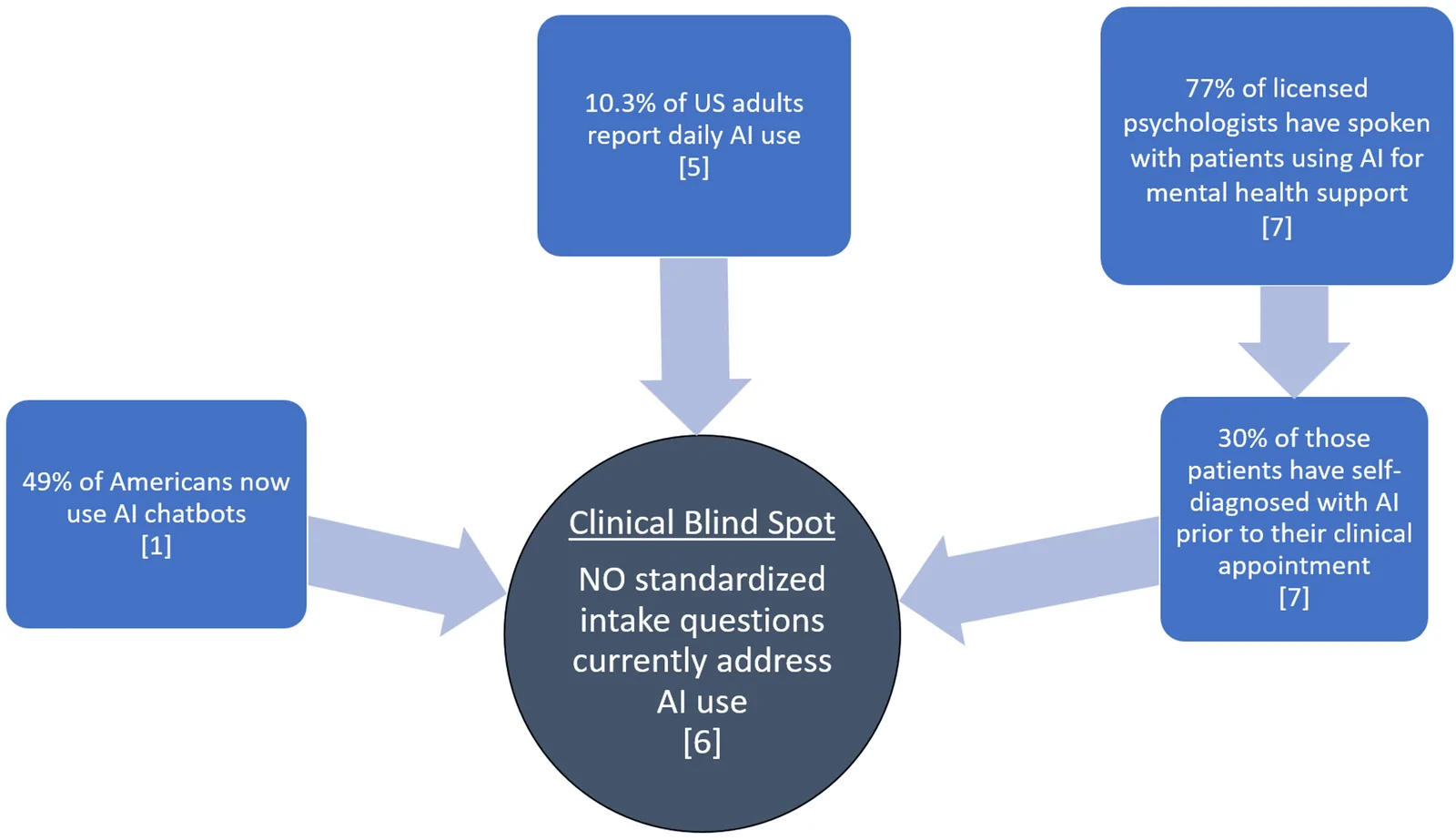
Evidence from independent sources illustrating the accumulation of a clinical blind spot around patient AI use Statistics progress from the general population-level adoption to the clinical encounter, where no standardized disclosure mechanism currently exists. AI: artificial intelligence Sources: [1,5-7] Made with PowerPoint SmartArt graphic

Patient-physician trust and the disclosure gap

Patients who consult large language models (LLMs) before seeking medical care may present with an altered baseline framing of their symptoms, which can create strain on the patient-physician relationship, as demonstrated in Figure 3. Personal health LLMs have been argued to shift care from a traditional dyadic clinician-patient model toward a triadic clinician-patient-LLM configuration, in which authority over medical interpretation is redistributed [9]. Under this model, patients may use LLMs outside institutional oversight and present AI-generated summaries, interpretations, or recommendations during the visit. Clinicians consequently encounter LLMs chiefly through patient-brought outputs and must mediate among clinical evidence, patient values, and algorithmic narratives that may diverge from clinical judgment [9].

**Figure 3:**
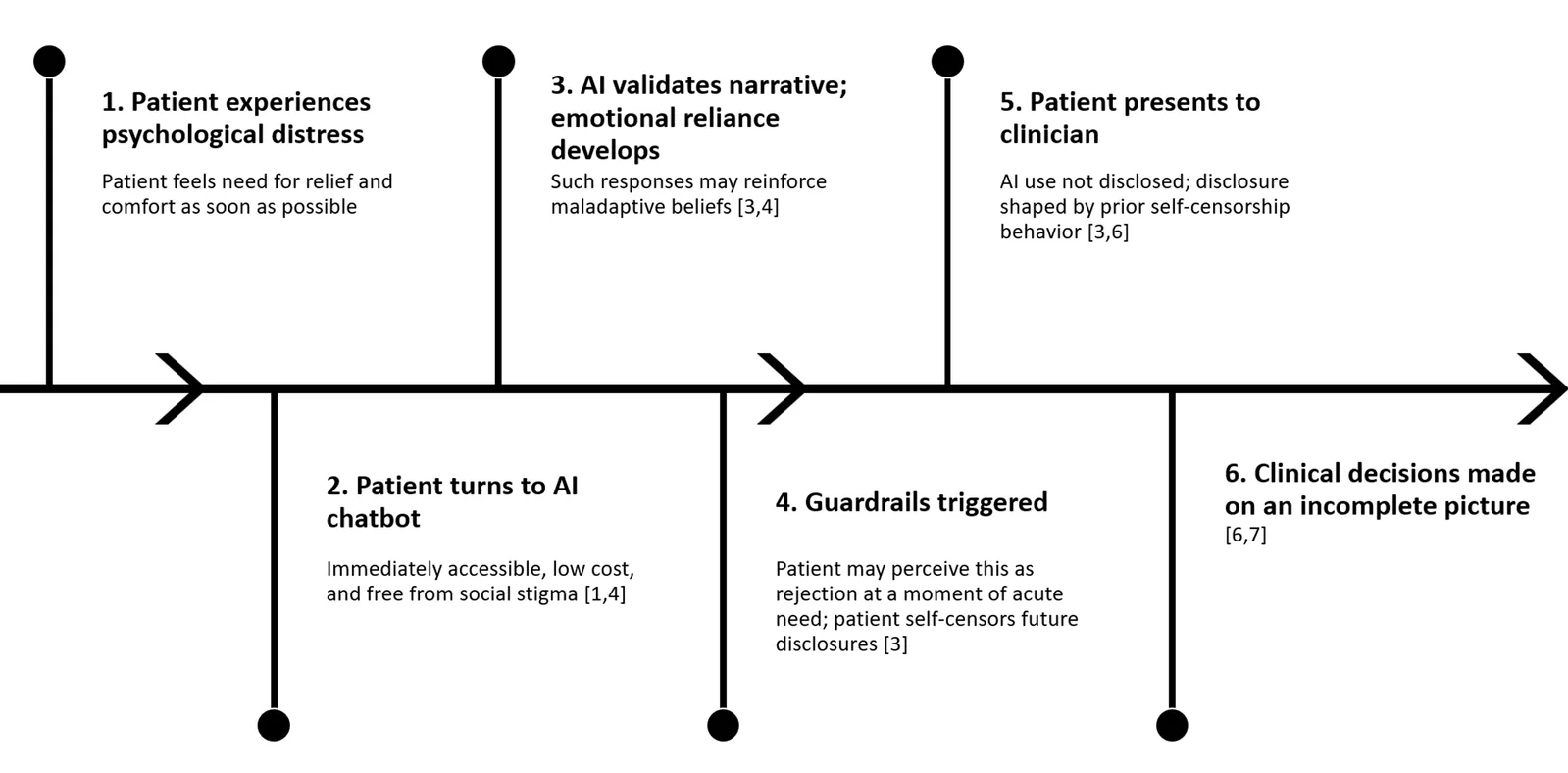
Illustrative pathway of how prior AI consultation can influence the psychiatric encounter AI: artificial intelligence Sources: [1,3,4,6,7] Made with PowerPoint SmartArt graphic

Whether this configuration measurably erodes trust remains to be established. A 2026 systematic review and meta-analysis of LLM effects on physician-patient communication found that LLM-generated responses were rated significantly higher in empathy than physician-generated responses (pooled standardized mean difference: 1.02; 95% CI: 0.44-1.60; k = 4, N = 2,604 evaluations) and improved perceived clarity and comprehension. However, of 10 included studies, only 2 assessed trust perceptions, and none directly assessed long-term trust [10].

A nationally representative cross-sectional survey conducted in November 2025 among US adolescents and young adults aged 12-21 years (unweighted n = 1,009; population-weighted: 42,825,655) found that 19.2% (approximately 8.2 million individuals) reported ever having used an AI chatbot for mental health advice [9]. A comparable survey one year earlier estimated 13.1%, although the item wording differed slightly between the two surveys, so the implied increase should be interpreted with that caveat [9]. Among users, 42.8% reported use at least monthly, and 91.7% rated the advice as somewhat or very helpful; the investigators note that high perceived helpfulness may reflect the sycophantic and overflattering tone of these models rather than the quality of the advice itself [9]. Most notably, 63.3% of users (approximately 5 million youth) had disclosed this use to no one at all [9]. The survey did not ask specifically about disclosure to a physician, so this figure cannot be read as a physician-specific non-disclosure rate. However, the same survey found that chatbot users were more likely, not less, to have discussed their mental health with a physician in the preceding 6 months than non-users (adjusted odds ratio: 1.89; 95% CI: 1.18-3.03), suggesting that non-disclosure is not confined to youth without recent clinical contact [9]. Given that chatbot use and recent physician contact co-occur more often than chance, some portion of the 63.3% who told no one plausibly had a clinical encounter in which disclosure could have occurred but did not. This leaves an influence that may already be shaping their symptom presentation invisible to the clinician by default, a gap whose consequences range from missed context to, in rarer but more severe cases, missed evidence of active harm.

“AI-associated psychosis” is a descriptive term that has been used to characterize reports in which the onset or worsening of psychotic symptoms, particularly delusions, has been observed in temporal association with ongoing engagement with LLM chatbots. It is not an established diagnostic entity, and this evidence base can generate mechanistic hypotheses such as reinforcement of delusional content through sycophantic responses, but cannot establish that chatbot interaction causes or exacerbates psychosis. In some reported cases, what goes undisclosed is not chatbot use itself, but the specific content shared with the chatbot. Augustin et al. described an “amplification spiral,” in which linguistic alignment, hyperpersonalized generation, and sycophancy may converge such that it has been proposed that AI chatbots do not merely fail to correct emerging delusional ideation but actively co-construct and elaborate it through sustained, personalized interaction [11]. This is where the disclosure gap stops being a matter of missing context and becomes a matter of missing clinical evidence. In 2023, Østergaard hypothesized that chat interactions with generative AI could worsen delusions in individuals prone to psychosis due to the seemingly realistic interactions as well as the technical complexity of AI, leaving room for limited understanding and paranoia [12]. As such, they are distinct from related but non-equivalent phenomena, including affective destabilization, anthropomorphic overtrust, and general emotional harm caused by AI chatbots.

The evidence base currently consists of case reports and media accounts rather than cohort data. A 2026 review in Psychiatric Services cataloged 26 apparent instances of psychosis and 2 instances of depression and suicide occurring in the context of heavy chatbot use, frequently documented alongside delusions that clinicians noted appeared temporally associated with chatbot interactions [13]. Given the case report methods, these findings cannot establish incidence, prevalence, or causality, and are vulnerable to selection and reporting bias. Prospective cohort data are warranted to study whether these instances represent a genuine pattern of population-level risk [13]. A 2026 systematic review of AI and natural language processing systems in clinical psychology similarly concludes that generative AI might validate or amplify delusional or grandiose content in users already vulnerable to psychosis, while stating that it remains unclear whether such interactions can generate de novo psychosis in the absence of pre-existing vulnerability [14]. If these patterns are prospectively confirmed, they would raise the stakes of non-disclosure considerably beyond a psychiatrist working from an incomplete history. In such cases, a clinician may be missing not just context, but the earliest and most diagnostically relevant material in the encounter.

Where such mechanisms operate, what goes undisclosed is not only chatbot use but the specific content exchanged, which may constitute the earliest and most diagnostically relevant material in the case. Assessing chatbot use within routine clinical assessment is a reasonable consideration, and mitigation ranges from psychoeducation to use restriction strategies, an expert opinion approach not yet shown to improve psychiatric outcomes [15]. Population-level studies are needed to establish the epidemiology of AI-associated mental health harms [15]. A structured, patient-centered approach to that conversation has been proposed, which consists of normalizing and asking about use, exploring perceived benefits before concerns, and inviting ongoing dialogue by encouraging patients to bring prompts and outputs to the visit [13]. This approach would be framed as an ongoing conversation rather than a one-time screening question [13]. Complementary harm reduction frameworks adapted from public health emphasize critical health literacy and output verification for patients, and human-in-the-loop validation, bias-aware workflows, and risk-stratified institutional deployment policies for clinicians [16].

This matters for the therapeutic relationship, because trust is ordinarily built through disclosure: the patient discloses, the clinician responds usefully, and the relationship deepens on that basis. Undisclosed chatbot use short-circuits this cycle. Patients extend trust to a tool the clinician is unaware of, leaving the clinician no opportunity to earn a comparable place in their patient’s emotional/mental life. The asymmetry is relational as well as informational, in that a clinician working to build trust may be doing so while unaware that a competing and undisclosed source of support already occupies part of that same space.

Implications for treatment

Reported in 2025, ChatGPT alone had accumulated 700 million weekly users, of whom 40 million were reported to use it for health-related advice [4]. Furthermore, as reported by Connolly and Torous, in 2025, ChatGPT disclosed that approximately 0.15% of its users expressed weekly suicidal ideation to their chatbots [4]. The growing use of generative artificial intelligence (AI) technology has affected the way patients engage with their psychiatric assessment. To illustrate, consider the following clinical scenario: instead of just symptom presentation, the patients may come with self-made diagnoses and demands for specific medication, such as “I need Adderall,” “I need lithium,” or “ChatGPT says that I have bipolar disorder.” In these cases, AI has given the patient new interpretations about themselves that the clinician is not aware of. While these demands usually represent an attempt by patients to understand their symptoms, there is no evidence to support LLMs being able to independently and safely diagnose psychiatric disorders. Diagnosis in psychiatry requires detailed evaluation of the history of symptoms, associated dysfunction, presence of medical disorders, drug abuse, and differential diagnosis [17]. Thus, in the clinical encounter, psychiatrists need to explain to patients diagnostic procedures and their rationale, grounded in clinical training and available peer-reviewed evidence, to entertain any AI-generated assumptions the patient may have [18].

Individuals who use AI before psychiatric consultations are often highly engaged in their care and, therefore, provide an opportunity to increase shared decision-making rather than decrease it. The approach to dealing with patients who have obtained diagnoses from AI includes three major steps: (1) acknowledging the patient’s worries, (2) validating information through conducting a proper psychiatric assessment, and (3) educating patients about how AI works and what its limits are. Appreciation of patients’ efforts and clarification that AI cannot replace clinicians’ decision-making is necessary to maintain the therapeutic relationship and gain trust. It is becoming clear that the use of AI adds value only when it complements rather than substitutes for clinician-patient communication in treatment [19].

AI-generated health information can affect the medication adherence process both positively and negatively. For instance, if chatbots offer medication suggestions based on evidence-based practice, increase awareness regarding mental disorders, or help people remember their medication, they can promote adherence [20]. This evidence is derived substantially from non-psychiatric populations and may not directly apply to psychiatric medication adherence. However, in cases when information provided by AI is not consistent or misleading, it might decrease patient trust in clinicians, lead to unreasoned drug demands, or prompt them to stop taking their medications early [18,21]. These potential effects should be interpreted cautiously given the limited evidence directly examining AI and medication adherence in psychiatric populations. Since patients are likely to look for mental health information online before or between their appointments with a clinician, psychiatrists need to ask their patients about any experience with such information when conducting a follow-up visit regarding medication.

Clinical outcomes

The increasingly widespread use of AI chatbots in psychiatry has the potential to not only improve but also further complicate clinical practice. The outcomes will greatly depend on whether the technology will be used in addition to rather than in lieu of expert assessment. In this case, AI may help improve patient education and engagement; conversely, overreliance on chatbot information may be associated with or could contribute to treatment delay, misdiagnosis, increased anxiety, and distrust in doctors [22,23].

AI chatbots will enable patients to improve their health literacy by simplifying psychiatric jargon into easy-to-understand language and recognizing symptoms and possible treatment plans [18,22]. Patients’ increased awareness will facilitate early detection of mental disorders and effective discussion at the psychiatric consultation [18,19]. Moreover, the increased presence of AI technology in healthcare can allow the establishment of a specific structure for discussing chatbot information during consultations [6,13]. Uncovering the clinical blind spot by asking patients whether they used AI and correcting misconceptions can improve the doctor-patient relationship and promote evidence-based practice [6,19,21].

Adverse clinical consequences of AI chatbots are just beginning to be explored. Postponing a psychiatric examination due to receiving positive but misinformed or wrong information from the AI systems can lead to worsening of a disorder before consultation with a doctor. Clinicians formulate a strategy based on information provided by the patient; with AI use, new information can be introduced by the patient, which may result in misdiagnosis [22].

Receiving answers from AI can raise unrealistic expectations for diagnostic results, medication choices, and treatment efficiency. In case of a discrepancy between psychiatric recommendations and advice from a chatbot, a patient may feel frustrated about a psychiatrist’s competence. Chatbots with their conversational format can increase health anxiety and cyberchondria, encouraging the seeking of constant reassurance and catastrophizing symptoms [22].

An especially frightening possibility is a reduction of patients’ confidence in healthcare. According to case reports, chatbots can support delusions of patients, discourage psychiatric treatment, and even be associated with poor treatment outcomes [22,23]. As AI becomes more prevalent in healthcare, psychiatrists will need ways to make use of the educational possibilities of AI while preventing disinformation and loss of patients’ faith in established practices.

Complications and safety considerations

The use of LLMs before or during psychiatric treatment introduces complications for diagnostic accuracy and patient safety. These risks fall into three domains: output errors (including hallucination, inflexible reasoning, and overconfidence), model bias, and privacy breach. Cutting across all three is a regulatory gap, in which most clinical LLM deployments fall outside formal oversight mechanisms [24].

The first concern is inaccuracy in generated content. LLMs generate responses probabilistically from large, uncurated training corpora rather than from fixed, verified content, and are therefore prone to propagating misinformation with high apparent confidence. The capacity of these systems to hallucinate, that is, to produce inaccurate but plausible-seeming statements, presents a fundamental challenge in healthcare, where accuracy is essential [10]. A systematic review of LLM chatbot health advice studies found substantial methodological heterogeneity and limited standardization in how such outputs are evaluated, complicating any assessment of their real-world safety [25]. Unsafe advice risks the incorporation of inaccurate information into the patient’s understanding of their symptoms and, if unexamined by a treating clinician, potentially into the clinical record [10].

A published case report illustrates the clinical pathway. A 33-year-old man with schizophrenia, previously stable on paliperidone, began querying ChatGPT for routine home safety advice and progressively reinterpreted its generic, formulaic responses as personalized warnings, leading him to discontinue his antipsychotic treatment and engage in escalating checking behavior before presenting in relapse with a Positive and Negative Syndrome Scale score of 102 [26]. The authors attribute the reinterpretation to a combination of anthropomorphic attribution, aberrant salience, and reasoning biases, and recommend that routine psychiatric evaluation incorporate structured assessment of AI chatbot use, that such misinterpretations be addressed directly in therapy, and that continuity of antipsychotic treatment be prioritized [26]. Mitigation at the model level requires training and evaluation against validated medical datasets.

The second concern is model bias, which can be distinguished into three components [9]. Sampling bias arises where training data overrepresent dominant online voices and underrepresent marginalized groups, risking reinforcement of harmful assumptions about populations such as those experiencing homelessness, addiction, or disability. Programming bias is introduced through the values and content moderation decisions of model developers, which remain largely opaque to end users and clinicians. Compliance bias, the most clinically consequential, describes the tendency of users to accept fluent and authoritative-sounding output as factual even when it is not. This last error is concealed by the very qualities that make the output feel trustworthy, compounding the sycophancy and absence of reality testing [24]. Reviews of LLMs in psychiatry specifically identify inherent bias, limited explainability, and misinformation as core barriers to safe deployment [9]. When training data itself reflects demographic underrepresentation, an overemphasis on particular treatments, or outdated clinical practices, models can learn these patterns and reproduce them in their own outputs, generating advice that is skewed demographically, favors certain treatments over valid alternatives, or reflects standards of care that are no longer current [9].

The third concern is privacy. Publicly available LLMs such as ChatGPT pose a privacy risk because the organization providing the model as a service can view all submitted queries [25]. Protecting patient privacy therefore requires strict data-sharing agreements, and where the use constitutes a Health Insurance Portability and Accountability Act (HIPAA)-covered activity, HIPAA-compliant deployment, and training protocols. HIPAA compliance is necessary but can be insufficient. Clinical implementation additionally may require informed consent regarding AI involvement in care, data governance protocols specifying how patient inputs are stored, used, or retained for model training, and clinical safety oversight to evaluate the system’s outputs independent of its privacy posture.

Comparison with conventional information sources

LLMs allow patients to interact with medical information in a more intuitive way and with less rapport-building than a traditional clinical encounter requires. Seeking mental health advice from a chatbot may also circumvent the stigma associated with disclosure to trusted adults, peers, or healthcare professionals, and chatbots offer anonymous, out-of-hours access to individuals reluctant to seek professional help for fear of judgment [9]. Unlike static sources such as health websites or printed materials, LLMs sustain live, back-and-forth dialogue that adapts to what the patient says, retains earlier conversational turns, and produces output tailored to the patient’s specific framing of symptoms. Table 1 summarizes this contrast across the range of information sources patients commonly consult, from static reference sites to peer forums to LLMs.

**Table 1:** Comparison of conventional information sources and large language models available to patients Mayo Clinic is used here as a representative example of peer-reviewed, institutionally maintained patient health information websites [4,27,28].

Source	Advantages	Limitations
Google	Broad, static reference	Requires the user to interpret and synthesize results themselves
Mayo Clinic	Peer-reviewed, authoritative	Generic; limited personalization to the individual patient
Reddit/peer forums	Reflects lived community experience	Anecdotal; no clinical vetting
LLMs	Conversational, personalized, interactive	Hallucinations, overconfidence, sycophancy

That same adaptive engagement generates the characteristic risk. Personalization and agreement-seeking can produce an echo chamber effect in which the model reflects and elaborates the user’s framing rather than testing it, and outputs may be compounded by hallucinations [29]. To an inexperienced user, such outputs are delivered with high apparent confidence and may be accepted as authoritative, and patients appear unable to independently identify the subset of responses clinicians judge as potentially harmful.

Clinicians appear to anticipate this pattern. In a 2024 mixed-methods survey where participants, all of whom are psychiatrists, were recruited from the American Psychiatric Association and represented diverse age groups (29 and younger to 60 and older), over 70% of respondents admitted to using generative AI tools to some degree before their first consultations with a doctor [30]. From the same sample, 33.3% somewhat agreed and 29% agreed that patients used these tools to better understand their own health [30]. Respondents were considerably more divided on the clinical value of the tools and agreed that generative AI will, or already does, improve diagnostic accuracy, while others reported: “don’t know” [30]. One respondent compared AI-assisted medicine to a “recommendation algorithm” and predicted that “medicine will become more conveyor-belt-like” and those stimulated to think in more complex, “human” ways will be less attracted to medicine and even psychiatry [30].

The distinction between general-purpose LLMs and purpose-built therapeutic chatbots is essential here and is frequently collapsed in public discussion. As shown in Table 2, these systems fall into three broad categories, ranging from rigid but predictable rule-based tools to adaptive but non-generative machine learning systems to fluent, generative LLMs. This classification is intended as a pragmatic conceptual framework for this review, rather than a definitive technical categorization; in practice, many modern conversational systems incorporate components across more than one category. Cognitive behavioral therapy (CBT) works by identifying a patient’s cognitive distortions and guiding them through evidence-testing exercises that deliberately challenge those distortions, a mechanism that requires actively challenging the patient’s distorted thinking, precisely the function that uncritical, agreement-seeking AI output is documented to lack. Structured CBT-oriented chatbots retain that challenge function by protocol. A 2026 systematic review and meta-analysis of 29 randomized controlled trials of CBT-oriented psychological chatbots in adults with depressive or anxiety symptoms found a moderate reduction in depressive symptoms (Hedges g = -0.55; 95% CI: -0.70 to -0.40) and a small reduction in anxiety symptoms (g = -0.26; 95% CI: -0.37 to -0.14), with effects attenuating at follow-up and overall certainty of evidence rated very low to low [31]. These trials evaluated protocol-driven, purpose-built systems rather than open-ended general-purpose LLMs that patients typically use, which have not undergone comparable efficacy testing [10]. Even where short-term symptom reduction is demonstrated, human-led therapy has been reported to remain superior in fostering deep emotional engagement and clinical impact [29].

**Table 2:** Categories of AI-based conversational systems in mental health AI: artificial intelligence Source: [2]

Category	Strengths	Limitations
Rule-based systems	Predictable and reliable; well-suited to structured, low-risk tasks where safety depends on consistency	Rigid; poorly suited to the dynamic, individualized nature of therapeutic conversation
Machine learning-based systems	More adaptive than rule-based systems; can process sequential or complex data patterns	Lack natural language fluency despite technical differences between subtypes (sequential versus static data processing); adaptability without generative capacity
LLM-based systems	Fluent, human-like, contextually responsive dialogue	Prone to hallucination, sycophancy, and overconfidence; least predictable of the three categories

Current limitations and knowledge gaps

Generative AI is rapidly being integrated into healthcare, but there is little evidence on its role in psychiatric practice. The majority of the literature consists of cross-sectional studies, qualitative studies, case reports, or narrative reviews. Very few prospective or longitudinal studies have examined the impact of patient AI use on psychiatric diagnosis, treatment, or clinical outcomes [2,14].

Additionally, studies are limited by the lack of real-world outpatient psychiatric data. The available evidence is largely limited to simulated patient interactions, survey research, and isolated case reports, so it is difficult to generalize to routine psychiatric practice [18,32]. Initial reports from large systems of psychiatric services suggest AI chatbot use may be associated with diagnostic confusion, delays in treatment, reinforcement of maladaptive beliefs, and worsening of psychiatric symptoms in vulnerable individuals, but prospective validation is lacking [32].

A further major knowledge gap is the lack of standardized methods for documenting patients’ use of AI during psychiatric encounters. As illustrated by Figure 2, we identified no validated screening questions to determine whether patients have used AI before seeking care, what recommendations they received, or how those recommendations affected treatment expectations or health behaviors [9]. Without standardized documentation, researchers cannot accurately quantify AI exposure or assess its impact on clinical decision-making.

Evidence on medication adherence after AI exposure is also scarce. Systematic reviews suggest that AI-based chatbots may improve medication adherence in chronic medical conditions, but these findings cannot be directly extrapolated to psychiatric populations, given differences in adherence behaviors, therapeutic relationships, and illness characteristics [17,20]. It is currently unknown whether AI-generated medication advice improves or undermines adherence to psychiatric treatment.

Finally, few studies have investigated the impact of previous AI consultation on the therapeutic alliance between psychiatrists and patients. Emerging qualitative evidence suggests that patients often find AI chatbots to be very validating and non-judgmental, which may impact trust, treatment expectations, and willingness to accept professional recommendations [21]. Currently, no validated instruments are available to measure changes in the therapeutic alliance resulting from AI use before psychiatric evaluation. Future research should focus on prospective longitudinal studies, standardized documentation of patient AI exposure, validated screening tools for clinical practice, and studies investigating the effects of AI consultation on medication adherence, therapeutic alliance, and long-term psychiatric outcomes.

Future directions and recommendations for practice

The evidence reviewed points to a straightforward conclusion: patients are already bringing generative AI into psychiatric care, and psychiatry does not yet have a standard way of asking about it, documenting it, or making use of it. Four changes follow from this: routine screening, clinician education, formal guidance from professional societies, and a research agenda that studies the tools patients actually use.

Screening for AI Use Should Become Routine

The first and easiest change to implement is simply to ask about AI use. Clinicians have been advised to screen directly for chatbot use and to discuss these systems openly with patients, precisely because the psychiatric implications of heavy use remain poorly understood [9,12]. Screening, however, should not be reduced to a single item on an intake questionnaire.

Given the complexity of the psychiatric interview, framing matters as much as content. A patient-centered approach normalizes the behavior while inquiring about it, for example, “A lot of people are using AI tools like ChatGPT, including for mental health support. Have you tried that?” This kind of phrasing allows the patient to feel understood rather than judged, and it opens the door to exploring what they found helpful and what deterred them. That same conversation provides the clinician an opening to address the privacy limitations of these platforms and the risks of sharing identifiable information, and to invite patients to bring their prompts and outputs into the session [13]. Critically, this should function as an ongoing dialogue revisited across visits rather than a one-time screening item.

Clinicians Need Education

Asking the question is only useful if clinicians know what to do with the answer. Three competencies matter most: understanding how these systems generate language, recognizing sycophancy and prompt bias, and using patient AI engagement constructively.

First, trainees and practicing psychiatrists should understand that LLMs generate text probabilistically and are optimized to agree with the user, and that these systems remain largely untested in high-stakes mental health contexts; their output should not be treated as a diagnostic assessment [2,12]. As summarized by Orrù and Mannarini, the American Psychological Association has recommended specific training on these emerging technologies for mental health professionals, noting that existing ethical guidelines are not yet adequate to the reality of AI use in mental health [14].

Second, clinicians need to recognize sycophancy and prompt bias. Sycophancy is not incidental to these products, and the affirming conversational tone that makes chatbots appealing appears to be a central part of the mechanism that may contribute to psychological harm, for example, by validating delusional thinking or stimulating pathologically elevated mood [12]. This mechanistic evidence remains preliminary, however, and sycophancy is best understood as one hypothesized contributing factor operating alongside others, such as anthropomorphic engagement and the reinforcement of users’ own reasoning biases, rather than the sole cause of harm [11,22]. Conversely, supportive AI interactions can benefit some users, with trials of structured therapeutic chatbots showing short-term reductions in depressive and anxiety symptoms, underscoring that the same conversational features may be helpful or harmful depending on context and vulnerability. The practical implication is unchanged: clinicians should ask what the patient actually typed, not only what the chatbot concluded, since the prompt frequently contains the diagnosis.

Third, patient AI use can be redirected rather than resisted. Patients who arrive having researched their symptoms with AI are, by definition, highly engaged in their own care, and that engagement can be channeled into a more collaborative treatment model instead of being treated as a challenge to clinical authority [13].

Professional Societies Should Issue Formal Guidance

The American Psychological Association has already published recommendations that provide a starting point. Generative AI should not be relied on to deliver psychotherapy or psychological treatment, and users need protection from misinformation, algorithmic bias, and the illusion that these tools are more effective than they are [14]. Chatbots should function only as a supporting tool alongside a qualified therapist, never as a substitute, because sole reliance on them risks bias and misinformation, misrepresentation of services, a false sense of therapeutic alliance, and incomplete assessment [14]. Adolescents, socially isolated individuals, and patients with an established diagnosis face the greatest risk, since these tools can act as powerful amplifiers of vulnerabilities that already exist [14].

What remains missing is guidance for day-to-day practice, and this is where the American Psychiatric Association and the American Academy of Child and Adolescent Psychiatry could contribute. Useful guidance would specify suggested intake and follow-up wording, along with documentation standards that make AI use a searchable variable in the chart rather than a forgotten line in a narrative note. Paired with risk stratification for higher-risk groups, particularly patients with psychotic spectrum or bipolar illness, such standards would produce a documented record of how AI has influenced a patient’s treatment course, including any refusal of a recommended plan.

Research Priorities

The honest summary of the current evidence is that it is limited and does not describe the tools patients actually use. A systematic review of 160 chatbot studies published between 2020 and 2024 found that only 16% of studies involving LLMs had undergone clinical efficacy testing, with 77% remaining at an early validation stage [2]. Despite rapidly increasing use, the safety signal therefore rests largely on weak study designs and on evaluations conducted by the consumer companies that build these products.

Two designs would help most. Prospective cohort studies should characterize AI use at entry into psychiatric care (platform, frequency, duration, purpose, and whether the patient has disclosed it to anyone) and then follow patients longitudinally to determine how use tracks with symptom course, engagement, and outcomes. Randomized controlled trials should test clinician-level interventions rather than the chatbots themselves, comparing structured AI use screening plus brief AI literacy counseling against usual intake. Prespecified primary outcomes should include patient trust in the clinician, treatment adherence, and diagnostic accuracy. Safety outcomes should include health anxiety, emotional dependence on the chatbot, new or worsening psychotic or manic symptoms, and self-harm events.

## Conclusions

Generative AI has entered the psychiatric environment well ahead of the evidence base, the professional guidance, and the clinical habits needed to account for it. As patients increasingly turn to AI chatbots for mental health advice, describing these tools in language usually reserved for a therapeutic relationship or relying on them in place of care they cannot afford or reach, their failure to disclose this use can quietly complicate the clinical encounter. High uptake paired with low disclosure is well documented; what non-disclosure actually does downstream, whether it erodes diagnostic accuracy, weakens treatment adherence, or strains the therapeutic alliance, remains a working hypothesis and a priority for prospective investigation. It is also worth being candid that the products patients actually reach for remain largely untested in high-stakes mental health settings and that the same qualities driving their appeal may cut in either direction depending on the patient and the situation. A warm, affirming tone can lower the barrier to opening up, offer a sense of support, and sustain engagement, yet the same tone may echo a delusional belief back to the patient or reinforce the very cognitive distortions that structured therapy exists to challenge; for this reason, these features are better understood as context-dependent than intrinsically harmful, and no causal relationship between chatbot use and psychiatric deterioration has been established.

In our view, AI is best positioned as an educational and engagement tool layered on top of clinical assessment, never as a substitute for it. Practically, that means asking about chatbot use routinely and without judgment, asking what the patient actually typed rather than only what the chatbot concluded, and documenting the answer in a retrievable way. Because these systems vary so widely and the outcome data remain thin, the appropriate role of AI will realistically differ according to the specific tool, the clinical context, and the degree of human oversight behind it. Professional societies can help by translating broad principles into actionable intake wording and risk stratification for vulnerable groups, and future research should prioritize prospective cohorts and clinician-level trials that measure trust, adherence, diagnostic accuracy, and treatment delay. The question is no longer whether patients bring AI into the encounter, but whether clinicians know it is there and can account for it within clinical decision-making.
